# An Evaluation of Initial Vancomycin Dosing in Infants, Children, and Adolescents

**DOI:** 10.1155/2011/470364

**Published:** 2011-10-23

**Authors:** Laura Broome, Tsz-Yin So

**Affiliations:** ^1^Department of Pharmacy, Moses H. Cone Memorial Hospital, Greensboro, NC 27401, USA; ^2^Department of Pharmacy, Moses H. Cone Memorial Hospital, 1200 North Elm Street, Greensboro, NC 27401-1020, USA

## Abstract

*Background*. The pharmacokinetics of many medications change as we age, thus most would assume dosing strategies would adjust for these changes. The objective of this study is to evaluate the initial vancomycin dosing in three pediatric age groups based on measured serum trough concentrations. *Methodology*. This retrospective database review included patients aged from 1 month to 18 years old admitted to the Moses H. Cone Memorial Hospital. Patients had to have received vancomycin dosed at 15 mg/kg every 8 hours with an appropriately measured trough concentration. The primary outcome was to determine the percentage of patients in 3 pediatric age groups achieving therapeutic trough concentrations with the initial vancomycin dosing regimen. *Results*. Twenty-five patients were included in the study. None of the patients had therapeutic trough concentrations after receiving vancomycin 15 mg/kg every 8 hours. Only one patient had a supratherapeutic level, while all of the other patients had levels less than 10 mcg/mL. *Conclusions*. Vancomycin 15 mg/kg every 8 hours did not provide therapeutic serum trough concentrations for any pediatric age groups. Higher doses and/or more frequent dosing regimens need to be evaluated for each age group to determine the most appropriate strategies for producing therapeutic trough concentrations.

## 1. Introduction

The pharmacokinetics of many medications change as we age, dictating the need for dosing strategies that adjust for these changes. One such example is vancomycin. The half-life of vancomycin ranges from approximately 2 to 10 hours depending on age (Table 1) [1]. Focusing on patients aged 3 months to 4 years old compared to those older than 4 years, there is an approximately twofold difference in their half-lives, which can lead to a huge difference in the dosing strategies of vancomycin.

With these age-related differences in pharmacokinetics, one would expect dosing strategies to be adjusted. However, dosing of vancomycin in the pediatric population, excluding neonates, is not age-specific (i.e., infants, children, and adolescents), failing to adjust for the variability in pharmacokinetics among pediatric age groups (Table 2) [1, 2].

Previous studies have shown that higher doses and more frequent dosing strategies are needed to produce therapeutic concentrations. Glover and colleagues evaluated the dosing of vancomycin in 76 pediatric intensive care unit (PICU) patients aged from 1 month to 18 years old [3]. Vancomycin 20 mg/kg given every 8 hours was needed to obtain therapeutic concentrations. In another retrospective database review, Benner and colleagues evaluated several vancomycin dosing schemes in 357 patients aged from 1 month to 18 years old [4]. Vancomycin 10 mg/kg every 6 to 8 hours was compared to vancomycin 15 to 20 mg/kg every 6 to 8 hours. The latter dosing strategies were needed to produce more therapeutic levels. 

At the Moses H. Cone Memorial Hospital, the standard pediatric dosing protocol ordered by the physicians for vancomycin is 15 mg/kg every 8 hours. The objective of this study is to assess the need for a revision to this initial vancomycin dosing strategy in three pediatric age groups. To our knowledge, there are not any other studies available that have evaluated vancomycin dosing strategies based on different pediatric age groups. We hypothesize that patients falling into the infant and adolescent age groups would potentially still reach goal serum trough levels, while those in the child age group would need higher starting doses and/or more frequent dosing regimens.

## 2. Materials and Methods

This retrospective study reviewed data from patients admitted to the Moses H. Cone Memorial Hospital general pediatric unit and PICU between 2006 and 2010. Patients were included if the ages were from 1 month to 18 years old, received vancomycin 15 mg/kg every 8 hours, and had a respective trough concentration measured appropriately, defined as a serum level drawn approximately 30 minutes before the third or subsequent doses. Patients were excluded if they had poor renal function, defined as an increase in serum creatinine by 50 percent or more from their baseline or normal serum creatinine for their age. Neonates were also excluded from the study. 

Electronic medical records were reviewed, and data collected include the initial vancomycin dose and respective serum trough concentration, timing of the trough concentration, patient demographics, and renal function.

The primary outcome was the percentage of patients achieving goal serum trough concentrations defined as 10 to 15 mcg/mL for cellulitis and 15 to 20 mcg/mL for bacteremia, pneumonia, meningitis, osteomyelitis, and endocarditis. Secondary outcomes included the percentage of patients with subtherapeutic and supratherapeutic trough concentrations as well as the indication of vancomycin. Patients were divided into three groups based on their age for analysis. Infants were defined as ages from 1 to 23 months old, children from 2 to 12 years old, and adolescents from 13 to 18 years old [5]. Demographics and the primary outcome were analyzed using descriptive statistics, while the secondary outcomes were analyzed by the Fisher's exact test. The study was approved by the Institutional Review Board of the Moses Cone Health System.

## 3. Results

Of the 50 patients' medical records reviewed, only 25 were included in the analysis due to exclusions. Eighteen patients had been started on vancomycin using different dosing strategies other than 15 mg/kg every 8 hours. Four patients were excluded due to inappropriate trough timing, in which the trough concentration was charted as being measured after the next dose of vancomycin. Other patients who were excluded consist of one patient being less than one month old, one had no weight documented, and three had documented acute renal failure, of which 2 were appropriately started on a different dosing regimen. 

Baseline demographics of the three patient groups were similar. The majority of patients were female and Caucasian in the infant and child age groups, while all the patients in the adolescent group were male and evenly divided between Caucasians and African Americans (Table 3). None of the patients in the cohort had cystic fibrosis or any past medical histories that could have increased their volume of distribution. Most of the patients had their trough concentrations collected with the third or fourth doses of vancomycin and were on vancomycin for less than one week.

In regards to the primary outcome, none of the patients achieved goal serum trough concentrations with vancomycin dosed at 15 mg/kg every 8 hours (Table 4). The majority of the trough concentrations were subtherapeutic, with only one infant having a supratherapeutic level (Table 5). This patient was also on potentially nephrotoxic agents, including gentamicin and furosemide. 

The serum trough concentrations of the infants were more likely to fall between 5 to 10 mcg/mL (55.6% in the infant group compared to 41.7% in the child age group, *P* = 0.86), while those in the child age group had more levels less than 5 mcg/mL (41.7% in the infant group compared to 58.3% in the child age group, *P* = 0.86) (Table 6). The primary indication of vancomycin was cellulitis (Table 7). Other indications of vancomycin were pneumonia, meningitis, and bacteremia.

## 4. Discussion

Vancomycin is an antimicrobial indicated in patients suffering from infections with methicillin-resistant Gram-positive pathogens [1]. It is usually used as the empiric therapy if the proportion of methicillin-resistant Gram-positive pathogens is significant (>5%) in the patient population, or if the patients are suspected to be suffering from serious infections (e.g., bacteremia), or if other antimicrobials like clindamycin cannot effectively combat the infection believed to be caused by methicillin-resistant Gram-positive pathogens. At our institution, vancomycin is only utilized when the latter two incidences occur. Our drug of choice for less serious infections with methicillin-resistant Gram-positive pathogens is clindamycin, since such pathogens have a sensitivity of approximately 95% to clindamycin in our pediatric population. 

Many previous studies targeted vancomycin trough concentrations ranging from 5 to 15 mcg/mL, which is lower than the new recommendations by the American Society of Health System Pharmacists, the Infectious Diseases Society of America, and the Society of Infectious Disease Pharmacists [6]. Due to the rise in the mean inhibitory concentration (MIC), it is recommended that vancomycin trough concentrations should be maintained at least above 10 mcg/mL for less severe infection like cellulitis and between 15 and 20 mcg/mL for more complicated infections like bacteremia, pneumonia, meningitis, osteomyelitis, and endocarditis. These recommendations are primarily for the adult population, but they can probably be extrapolated to pediatric patients. 

Our institution's standard physician vancomycin dosing protocol of 15 mg/kg every 8 hours did not achieve the new goal serum trough concentrations for any of the pediatric age groups. Only one patient had a level greater than 10 mcg/mL, and the level was found to be supratherapeutic. Again, this patient was also on other agents likely to cause nephrotoxicity including furosemide and gentamicin. Previous studies have shown that the risk of nephrotoxicity while on vancomycin increases with concomitant use of other nephrotoxic agents like gentamicin, as well as an extended duration of therapy, leading to high concentrations of vancomycin and potential kidney damage [7].

In a study by Glover and colleagues, vancomycin 20 mg/kg every 8 hours produced a mean trough concentration of 7.8 mcg/mL, whereas vancomycin 15 to 20 mg/kg every 6 to 8 hours produced levels from 8 to 12 mcg/mL in a study by Benner et al. Only the vancomycin dosing regimens with the frequency of every 6 hours was able to produce trough concentrations greater than 10 mcg/mL. None of the dosing strategies in either trial were able to reach concentrations greater than 15 mcg/mL needed for more complicated infections per the new recommendations. Interestingly, the mean age for each study was 5 years old, falling into the shortest half-life group for vancomycin. 

In a newer study of 430 pediatric patients aged from 1 month to 18 years old, Eiland and colleagues found that vancomycin dosing strategies of 70 mg/kg/day and 85 mg/kg/day were needed to produce target serum trough concentrations of 10 mcg/mL and 15 mcg/mL, respectively [8]. Using the standard pediatric dosing regimens of 40 to 60 mg/kg/day only reached therapeutic levels 49 percent of the time based on the new higher serum trough concentration recommendations. The mean age of the patient population was 5.9 years, which also fell into the child age group.

In our study, those in the infant age group were more likely to have a trough level in the 5 to 10 mcg/mL range, while the patients in the child age group had more trough concentrations less than 5 mcg/mL. The adolescent group's trough concentrations were evenly divided between troughs less than 5 mcg/mL and 5 to 10 mcg/mL. However, due to the small sample size of adolescents, no conclusions can be drawn though one would expect based on the hypothesized adolescent half-life that their trough concentrations would be similar to those of the infant group. We expect that if our patient population had been larger, we might have been able to see more of a trend with each age group. Unfortunately, the sample size was small due to half of the patients being excluded. Originally, we had set out to collect and analyze more patient data, but due to the fact that all the patients' serum trough concentrations were subtherapeutic, data collection was stopped early. Other limitations to the study include its retrospective nature and the fact that it was conducted at a single center.

Nonetheless, it is very important to provide a vancomycin dosing regimen to patients that will lead to therapeutic concentration in a timely manner, since methicillin-resistant Gram-positive pathogens, especially *staphylococcus aureus*, have become more virulent in recent years. As a matter of fact, a study by Welsh and colleagues confirmed a high rate of vancomycin treatment failures (*∼*50%) in pediatric patients with methicillin-resistant *staphylococcus aureus* (MRSA) bacteremia [9]. This failure might have been due to either subtherapeutic vancomycin trough concentrations or the development of vancomycin-resistant MRSA. The latter is of a grave concern, since there are limited data on the use of other more powerful antimicrobials treating methicillin-resistant or vancomycin-resistant Gram-positive pathogens in the pediatric population. One potential alternative to vancomycin is linezolid. Numerous reports and studies exist that describe the safe and effective use of linezolid in the pediatric population [10–12]; however, there is not any comparative study between these two agents (i.e., vancomycin and linezolid) in pediatric patients with MRSA infections. If this study is to be conducted, the higher vancomycin dosing strategies need to be considered in the design in order to reach therapeutic concentration.

## 5. Conclusion

The traditional dosing schemes of vancomycin, including our standard physician protocol of 15 mg/kg every 8 hours, most likely do not produce therapeutic trough concentrations in otherwise healthy patients with normal renal function. Our next step at the Moses H. Cone Memorial Hospital is to start dosing vancomycin at 20 mg/kg every 8 hours for patients in the infant and adolescent age groups, and vancomycin 15 mg/kg every 6 hours for patients in the child age group. However, for our pediatric patients with clinically significant comorbidities (e.g., congenital heart disease, nephrotic syndrome, and renal dysfunction), we will continue dosing vancomycin at 15 mg/kg every 8 hours. We then hope to evaluate these two dosing strategies in the future, as these doses and/or frequencies may need to be pushed even higher, especially for patients in the child age group due to their short half-life. Large randomized controlled trials are needed to determine the most appropriate dosing regimens of vancomycin in the pediatric population, and the differences in pharmacokinetics must be recognized in order to develop the best dosing for each patient.

## Figures and Tables

**Table 1:** Vancomycin half-life [1].

Age group	Half-life
Neonates	6–10 hours
3 months to 4 years old	4 hours
>4 years old	2.2–3 hours
Adolescents	Not well defined (hypothesized to be similar to an infant)
Adults	5–8 hours

**Table 2:** Vancomycin dosing regimens [1, 2].

Age group	Dosing Regimen
Neonates	10–15 mg/kg every 6–18 hours depending on PMA and PNA
Infants, children, and adolescents	10 mg/kg every 6 hours (traditional dosing) or 15–20 mg/kg every 6–8 hours (serious infection)
Adults	15–20 mg/kg every 8–12 hours
Moses H. Cone Hospital (pediatrics)	15 mg/kg every 8 hours

PMA: postmenstrual age; PNA: postnatal age.

**Table 3:** Demographics.

Parameters	Infant (*N* = 9)	Child (*N* = 12)	Adolescent (*N* = 4)
Room, No. (%)			
General Pediatric	6 (66.7)	12 (100)	4 (100)
PICU	3 (33.3)	—	—
Gender, No. (%)			
Male	4 (44.4)	5 (41.7)	4 (100)
Race, No. (%)			
Caucasian	4 (44.4)	7 (58.3)	2 (50)
African American	3 (33.3)	4 (33.3)	2 (50)
Hispanic	2 (22.2)	—	—
Asian	—	1 (8.3)	—
Mean age, yrs (range)	0.78 (1–23 months)	5.3 (2–12)	14.8 (13–17)
Mean initial creatinine level, mg/dL (range)	0.4 (0.3–0.5)	0. 4 (0.3–0.7)	0.6 (0.3–0.8)
Timing of level, No. (%)			
Before 3rd dose	4 (44.4)	6 (50)	3 (75)
Before 4th dose	3 (33.3)	5 (41.7)	—
Before >4th dose	2 (22.2)	1 (8.3)	1 (25)
Mean duration of therapy, days (range)	4.9 (1–21)	3.8 (1–16)	2.5 (1–4)

**Table 4:** Percentage of patients achieving goal vancomycin serum trough concentrations.

Age group	Patients, no.	%
Infant	9	0
Child	12	0
Adolescent	4	0

**Table 5:** Vancomycin trough concentrations.

Age group	Subtherapeutic, no. (%)	Supratherapeutic, no. (%)
Infant	8 (88.9)	1 (11.1)
Child	12 (100)	—
Adolescent	4 (100)	—

*P* = 0.52.

**Table 6:** Distribution of subtherapeutic vancomycin trough concentrations.

Age group	Trough concentrations (mcg/mL), no. (%)		
	<5	5–10	10–15
Infant	3 (33.3)	5 (55.6)	—
Child	7 (58.3)	5 (41.7)	—
Adolescent	2 (50)	2 (50)	—

*P* = 0.86.

**Table 7:** Indications of vancomycin.

Indication	Infant, no. (%)	Child, no. (%)	Adolescent, no. (%)
Cellulitis	4 (44.4)	5 (41.7)	2 (50)
Pneumonia	2 (22.2)	6 (50)	2 (50)
Meningitis	2 (22.2)	—	—
Bacteremia	1 (11.1)	1 (8.3)	—

*P* = 0.66.
